# Erosive Esophagitis Unveiled: A Retrospective Study of Epidemiology and Endoscopic Findings in Qassim, Saudi Arabia

**DOI:** 10.7759/cureus.93688

**Published:** 2025-10-02

**Authors:** Abdulmajeed Albarrak, Rana Alsamani, Raghad R Alwattban, Yasmeen A Alfouzan, Norah M Alharbi, Resheed Alkhiari, Ahmad Alshomar, Omar Almansour, Fayez S Alreshidi, Majid Alsahafi

**Affiliations:** 1 Department of Medicine, College of Medicine, Qassim University, Qassim, SAU; 2 Division of Gastroenterology, Department of Medicine, Dr. Suliman Al Habib Medical Group, Qassim, SAU; 3 Division of Gastroenterology, Department of Medicine, College of Medicine, King Saud University, Riyadh, SAU; 4 Department of Family and Community Medicine, College of Medicine, University of Hail, Hail, SAU; 5 Division of Gastroenterology, Department of Medicine, Faculty of Medicine, King Abdulaziz University, Jeddah, SAU

**Keywords:** erosive esophagitis, gastroesophageal reflux disease, gastrointestinal, hiatal hernia, prevalence

## Abstract

Background: Gastroesophageal reflux disease (GERD) is a common gastrointestinal condition caused by the reflux of gastric contents into the esophagus. Untreated GERD can lead to complications, including erosive esophagitis (EE), Barrett’s esophagus, and esophageal stricture. This study aimed to determine the prevalence of EE among patients undergoing endoscopy in Qassim, Saudi Arabia, and to identify associated risk factors.

Methods: We conducted a retrospective study at Habib Medical Group Hospital in Qassim, Saudi Arabia, including adult patients (≥18 years) who underwent upper gastrointestinal endoscopy between July 2021 and August 2022. Data were collected from electronic health records and included demographic information, body mass index (BMI), clinical presentation, endoscopy indication, and EE severity according to the Los Angeles (LA) classification. Associations between EE and potential predictors were assessed using chi-square testing and multivariate logistic regression. A p-value <0.05 was considered statistically significant. Data were analyzed using SPSS version 26 (IBM Corp., Armonk, NY, USA).

Results: A total of 1,010 patients were included. The prevalence of EE was 12.3% (124 patients). Males accounted for 65.3% of EE cases (p < 0.001). The majority of EE cases were mild, with 97 (78.2%) classified as LA grade A and 17 (13.7%) as grade B. Multivariate analysis revealed that obesity was a strong independent predictor of EE, with an adjusted odds ratio (AOR) of 9.834 (95% CI: 2.525-38.304; p = 0.001). The presence of a hiatal hernia was also significantly associated with EE (AOR = 7.365; 95% CI: 1.898-28.582; p = 0.004). Male sex did not remain significant after adjustment (p = 0.069).

Conclusions: The prevalence of EE in this study population was 12.3%, with most cases categorized as mild (LA-A). Higher BMI and the presence of a hiatal hernia were independent and significant predictors of EE, whereas male sex was not independently associated. These findings highlight the importance of addressing modifiable risk factors such as obesity in the prevention and management of EE.

## Introduction

Gastroesophageal reflux disease (GERD) is a prevalent medical condition characterized by the reflux of gastric acid into the esophagus. If left untreated, GERD can result in a range of complications. The potential outcomes of this condition vary from mild non-erosive esophagitis to more serious issues such as erosive esophagitis (EE) and Barrett's esophagus (BE) [1]. Several risk factors have been identified in relation to GERD, including smoking, excessive alcohol consumption, a high body mass index (BMI), hiatal hernia, pregnancy, and psychological stress [2].

Erosive and non-erosive reflux disease are types of GERD. Non-erosive reflux disease (NERD) is characterized by the absence of mucosal tears in the esophagus, while classic reflux symptoms are still present. Patients with EE, diagnosed endoscopically, have mucosal breaks in their lower esophagus [3]. Ulcers, bleeding, and strictures are the most common esophageal consequences of EE. Although deaths due to EE are extremely rare, these complications are associated with critical morbidity [4].

A 2012 study conducted in Taiwan revealed the prevalence of EE at 17.3% (352 out of 2040 participants). Among these cases, 71.6% were classified as grade A, 27.8% as grade B, 0.5% as grade C, and none as grade D, according to the Los Angeles (LA) classification [5-7]. In the same year, another study in Iran included 736 patients experiencing GERD symptoms, with 283 patients (38%) diagnosed with EE and 34 with BE [6]. Locally, a study in Riyadh, Saudi Arabia, showed an incidence of GERD of 45.4%, with higher prevalence among the elderly, obese individuals, and smokers [8].

This retrospective analytical study aimed to determine the prevalence of erosive esophagitis among patients undergoing endoscopy at a tertiary hospital in Saudi Arabia and to identify independent risk factors, including BMI, sex, age, hiatal hernia, and GERD symptoms then presenting multivariate regression findings on these factors.

## Materials and methods

Study design and setting

This retrospective study was conducted at Habib Medical Group Hospital in the Qassim region of Saudi Arabia.

Patient selection

All adult patients (≥18 years) who underwent upper gastrointestinal endoscopy between July 2021 and August 2022 were included. Pediatric patients and repeat endoscopies were excluded.

Data collection

Data were extracted from the structured electronic health records (EHRS) system. Variables collected included age, sex, BMI, main presenting symptoms, indication for endoscopy, post-endoscopy diagnosis, and the presence and severity of EE according to the LA classification system (grades A-D), where grade A is one (or more) mucosal break(s) ≤5 mm, not bridging the tops of mucosal folds; grade B is one (or more) mucosal break(s) >5 mm, not bridging the tops of mucosal folds; grade C is mucosal breaks bridging the tops of two or more mucosal folds, involving <75% of the esophageal circumference; and grade D is mucosal breaks involving ≥75% of the esophageal circumference.

Multivariate logistic regression analysis was performed to determine independent predictors of EE. Variables included in the model were BMI, sex, and presence of hiatal hernia.

Statistical analysis

Data are presented as frequencies and percentages. Chi-square tests were used to examine associations between categorical variables (e.g., gender, presence of hiatal hernia) and EE status. A multivariate logistic regression model was conducted to identify factors including BMI, sex, and presence of hiatal hernia independently associated with EE. Variables with p ≤ 0.05 in univariate analysis were included in the regression model. Adjusted odds ratios (AOR) with 95% confidence intervals (CI) were calculated, and statistical significance was set at p < 0.05. Statistical analyses were conducted using SPSS version 26 (IBM Corp., Armonk, NY, USA).

## Results

This study included 1,010 patients. As shown in Table 1, the most common age group was 21-40 years, comprising 485 patients (48.0%). Sex distribution was equal, with 505 males (50.0%) and 505 females (50.0%). Among patients with EE, 38 (30.6%) had a BMI below 25 kg/m². The overall prevalence of EE was 124 patients (12.3%), with grade A being the most common subtype (97 cases, 78.2%) according to the Los Angeles classification. Hiatal hernia was observed in 102 patients (10.1%), and pain was the most frequent indication for endoscopy (305 patients, 30.2%).

**Table 1:** Baseline characteristics of the patients (n = 1010) Data are presented as number (%).

Study variables		n (%)
Age group	<21 years	72 (07.1%)
	21–40 years	485 (48.0%)
	41–60 years	312 (30.9%)
	>60 years	141 (14.0%)
Sex	Male	505 (50.0%)
	Female	505 (50.0%)
BMI level ^(n = 124)^	<25 kg/m^2^	38 (30.6%)
	25–29 kg/m^2^	34 (27.4%)
	30–34 kg/m^2^	32 (25.8%)
	>34 kg/m^2^	20 (16.1%)
Erosive esophagitis		124 (12.3%)
Los angles grade^ (n = 124)^	Grade A	97 (78.2%)
	Grade B	17 (13.7%)
	Grade C	08 (06.5%)
	Grade D	02 (01.6%)
Hiatus hernia		102 (10.1%)
Indication	GERD	134 (13.3%)
	Dyspepsia	260 (25.7%)
	Pain	305 (30.2%)
	Dysphagia	94 (09.3%)
	Other	217 (21.5%)

As shown in Table 2, the prevalence of EE was significantly higher among male patients (P < 0.001), those with elevated BMI (P = 0.003), patients with a hiatal hernia (P < 0.001), and those diagnosed with GERD (P < 0.001)

**Table 2:** Relationship between erosive esophagitis and the baseline characteristics of the patients (n=1010) § P-value has been calculated using the chi square test. ** Significant at P≤0.05 level.

Factor		Erosive Esophagitis		P-value ^§^
		Yes N (%) (n=124)	No N (%) (n=886)	
Age group	≤40 years	60 (48.4%)	497 (56.1%)	0.106
	>40 years	64 (51.6%)	389 (43.9%)	
Sex	Male	81 (65.3%)	424 (47.9%)	<0.001 **
	Female	43 (34.7%)	462 (52.1%)	
BMI level ^(n=160)^	<30 kg/m^2^	74 (59.7%)	31 (86.1%)	0.003 **
	≥30 kg/m^2^	50 (40.3%)	5 (13.9%)	
Hiatus hernia	Yes	53 (42.7%)	49 (05.5%)	<0.001 **
	No	71 (57.3%)	837 (94.5%)	
Indication ^†^	GERD	32 (31.1%)	102 (14.8%)	<0.001 **
	Dyspepsia	25 (24.3%)	235 (34.1%)	
	Pain	32 (31.1%)	273 (39.6%)	
	Dysphagia	14 (13.6%)	80 (11.6%)	

The results of a multivariate regression analysis (Table 3) indicate that individuals with a higher BMI and the presence of a hiatal hernia were significant independent predictors of EE. Patients with an increased BMI were linked with a nearly tenfold higher likelihood of experiencing EE compared to those with a lower BMI (AOR = 9.834; 95% CI: 2.525-38.304; p = 0.001). Those with a hiatal hernia had a 7.4-fold increased risk compared to those without (AOR = 7.365; 95% CI: 1.898-28.582; p = 0.004). Pain appeared to be associated with a lower risk of EE; however, this association was borderline (AOR = 0.159; 95% CI: 0.025-0.999; p = 0.050) and should be interpreted with caution. On the other hand, when comparing individuals with symptoms of GERD to those with dyspepsia, the latter group showed an apparent reduction in EE risk; however, this association was not statistically significant (AOR = 0.607; 95% CI: 0.148-2.487; p = 0.488). Nevertheless, sex did not have a statistically significant impact following adjustment in the regression model (p = 0.069).

**Table 3:** Multivariate regression analysis of different variable versus EE AOR – Adjusted Odds Ratio; CI – Confidence Interval. ** Significant at P≤0.05 level.

Factor		AOR	95% CI	P-value
Sex	Male	Ref	-	-
	Female	0.403	0.152 – 1.072	0.069
BMI level ^(n=160)^	<30 kg/m^2^	Ref	-	-
	≥30 kg/m^2^	9.834	2.525 – 38.304	0.001 **
Hiatus hernia	Yes	7.365	1.898 – 28.582	0.004 **
	No	Ref	-	-
Indication ^†^	GERD	Ref	-	-
	Dyspepsia	0.607	0.148 – 2.487	0.488
	Pain	0.159	0.025 – 0.999	0.050 **
	Dysphagia	0.854	0.243 – 2.994	0.805

## Discussion

In this retrospective study of 1,010 adult patients undergoing upper gastrointestinal endoscopy in Qassim, Saudi Arabia, the prevalence of EE was 12.3% (124/1,010). Most EE cases were mild, with 97 (78.2%) classified as LA grade A, followed by 17 (13.7%) as grade B, eight (6.5%) as grade C, and two (1.6%) as grade D. Multivariate analysis revealed that patients with a BMI ≥30 kg/m² had a significantly higher risk of EE (adjusted odds ratio [AOR] = 9.834, 95% CI 2.525-38.304; p = 0.001), and those with hiatal hernia had a similarly elevated risk (AOR = 7.365, 95% CI 1.898-28.582; p = 0.004). Although male sex was significantly associated in univariate testing, this association did not remain significant after adjustment (AOR = 0.403, 95% CI 0.152-1.072; p = 0.069).

The strong association of obesity with EE can be explained both mechanically, through increased intra-abdominal pressure and transient lower esophageal sphincter relaxations, and metabolically, via inflammatory mediators that compromise esophageal mucosal integrity. Similarly, hiatal hernia predisposes to EE by disrupting the gastroesophageal junction and prolonging acid exposure. The predominance of LA-A disease in this cohort suggests that most patients are diagnosed at an early stage, possibly due to referral and surveillance practices.

Our findings are consistent with a large body of evidence identifying obesity and hiatal hernia as the principal determinants of EE. Witarto et al. [9] conducted a systematic review of 114 observational studies (759,000 participants) confirmed that obesity (OR = 1.78), central obesity (OR = 1.29), and hiatal hernia (OR = 4.07) are consistent risk factors for EE, directly supporting our results. Xie et al. [10] reported EE prevalence of 17.3% (352/2,040), identifying obesity and hiatal hernia as independent predictors, closely aligning with our observed associations. In Japan, Ohashi et al. [11] reported EE in 27.3% (118/433) and emphasized visceral obesity and hernia as main risks, while Koo et al. [12] demonstrated that abdominal obesity increases EE indirectly through hernia formation among Koreans. In the Philippines, Magallen et al. [13] showed EE was more common in obese GERD patients (52.3%) compared to non-obese (21.8%). Also, Sharara et al. [14] showed EE in 33.9% (82/242), with independent predictors including hiatal hernia, GERD questionnaire score, Hill grade, and tobacco use.

Recent publications provide further support. Chait [15] stressed that weight reduction and early identification of hiatal hernia remain essential for prevention. Kim et al. [16] linked metabolic deterioration and insulin resistance with higher EE risk, thereby connecting metabolic syndrome directly to reflux injury. Qi et al. [17] found that BMI correlated positively with hiatus size (r between 0.54 and 0.72, all P < 0.01). Although obese patients had larger hiatus regardless of reflux esophagitis or hiatal hernia status, the study supports the hypothesis that larger hiatus size associated with obesity may contribute to increased risk of esophageal injury through reflux exposure.

In contrast, Alsahafi et al. [18] studied 2,805 patients and reported a similar EE prevalence of 11% in Saudi Arabia, but obesity was not significant after adjustment, leaving hiatal hernia as the key independent factor. In Korea, Seo et al. [19] found an 8.8% prevalence (2,241/25,536) and identified BMI as predictive, but not male sex. Alzanbagi et al. [20] in Saudi Arabia showed EE prevalence of 30% five years after surgery. Univariate analysis associated pre‐laparoscopic sleeve gastrectomy BMI with EE (p = 0.038), though in their multivariate model, no factor remained statistically significant for EE.

This study has several limitations. Its retrospective design limits causal inference and is subject to information bias. Data on lifestyle factors (diet, smoking, alcohol), medication use, and H. pylori status were unavailable, which may confound associations. The study was conducted in a single private hospital, which may not represent the general Saudi population. The cross-sectional nature precludes assessment of long-term outcomes such as progression to Barrett’s esophagus or stricture.

Given these findings, weight management and early detection of hiatal hernia should be emphasized in clinical practice to reduce EE risk. Further multicenter prospective studies in Saudi Arabia are warranted, incorporating lifestyle, metabolic, and microbiological data to better clarify risk profiles and preventive strategies.

## Conclusions

The prevalence of EE in this Saudi cohort was 12.3%, predominantly mild (LA-A). Obesity and hiatal hernia emerged as independent predictors, whereas male sex did not remain significant after multivariate adjustment.
